# Breaking the stigma: the role of family support in hepatitis B management from a public health education perspective

**DOI:** 10.3389/fpubh.2026.1768182

**Published:** 2026-03-12

**Authors:** Lin Zhang, Junyi Chen, Guoxing Wu, Suhua Pang, Zhenjiang Zhang, Youde Yan

**Affiliations:** 1 Department of Infectious Diseases, Jiangsu Province (Suqian) Hospital, Suqian, Jiangsu, China; 2 The Suqian Clinical College of Xuzhou Medical University, Suqian, Jiangsu, China; 3 Department of Infectious Diseases, The First Affiliated Hospital of Nanjing Medical University, Nanjing, Jiangsu, China

**Keywords:** family support, family-centered care, hepatitis B, nursing, public health, stigma

## Abstract

Hepatitis B remains a major global public health challenge. Beyond its clinical burden, hepatitis B-related stigma persists as a multilevel social process embedded within relational and institutional structures. Despite advances in vaccination and antiviral therapy, individuals living with hepatitis B continue to experience psychological distress and social marginalization shaped by stigma. Family systems represent a central relational context in which infection identity is interpreted and negotiated. Through disclosure practices, identity organization, and responses to institutional pressures, families regulate the visibility and salience of infection identity. At the same time, stigma may disrupt these processes, weaken support structures, and undermine psychological well-being and treatment engagement. This narrative review reconceptualizes the interaction between family support and hepatitis B–related stigma as a dynamic pathway operating across individual, familial, and structural levels. It highlights how healthcare institutions—particularly nursing systems embedded in long-term disease management—can modulate stigma through structured communication, relational repositioning, and institutional risk navigation. These findings underscore the need for family-centered, nursing-integrated, and public health–aligned strategies that address stigma beyond individual cognition. Such an approach provides a structured foundation for sustainable stigma reduction and equity-oriented support for people living with hepatitis B.

## 1 Background

Hepatitis B remains a major global public health concern caused by infection with the hepatitis B virus (HBV). According to the World Health Organization (WHO), approximately 296 million people worldwide are living with chronic hepatitis B, with nearly 800,000 deaths annually attributable to complications such as cirrhosis and hepatocellular carcinoma (1). Despite widespread vaccination and effective antiviral therapies, hepatitis B–related mortality has changed little over the past two decades. Notably, only about 13% of individuals with HBV are aware of their infection identity, and access to diagnosis and long-term care remains limited, particularly in low- and middle-income countries. Stigma and discrimination represent substantial barriers to achieving the WHO goal of eliminating hepatitis B as a public health threat by 2030 (2, 3).

Unlike many chronic conditions, hepatitis B is associated with distinctive stigma-generating mechanisms rooted in its transmission patterns, sociocultural meanings, and policy histories. Vertical transmission in high-prevalence regions may generate intergenerational blame and maternal guilt. Concerns regarding disclosure, fertility, and transmission risk contribute to reproductive and marital stigma. In some settings, historical and ongoing employment screening practices reinforce structural discrimination, while the enduring “carrier” identity attached to chronic infection sustains labeling even in asymptomatic individuals (4, 5). Together, these mechanisms create a stigma ecology that is deeply embedded in family systems.

Family support occupies a central position in the lives of people living with hepatitis B, serving not only as emotional assistance but also as the primary relational context in which stigma is interpreted and negotiated. Individuals often experience dual pressures arising from broader social discrimination and intra-family tensions related to transmission, reproduction, and economic stability (6, 7). Stigma may amplify self-blame and misunderstandings regarding transmission routes, weakening trust, communication, and cohesion within family systems (8, 9). Such dynamics can undermine psychological well-being, treatment adherence, and long-term disease management.

Families themselves are shaped by external stigmatization, influencing disclosure patterns, caregiving roles, and decision-making processes (9). More than 60% of individuals with HBV reside in Southeast Asia and the Western Pacific, where stigma remains particularly visible, including in China (10). In high-prevalence contexts, cultural expectations surrounding lineage, reproduction, and social productivity may intensify family-level consequences of stigma. Understanding how hepatitis B–related stigma operates within family systems is therefore essential not only for clarifying psychosocial vulnerability but also for identifying modifiable pathways that require tailored public health and nursing responses (11).

This review synthesizes existing literature to examine how hepatitis B–specific stigma mechanisms interact with family systems across disease stages and sociocultural contexts. By situating family support within a broader stigma pathway framework, we aim to provide a structured conceptual basis for family-centered and nursing-integrated interventions in public health practice.

## 2 Family support

### 2.1 Conceptualization of family support

Family support refers to the multidimensional assistance provided by family members, encompassing emotional, informational, and practical domains (12). In the context of hepatitis B, family support serves not only as a fundamental resource for daily living but also as a critical factor influencing patients’ ability to cope with disease-related challenges, maintain treatment adherence, and sustain quality of life (13).

### 2.2 Modalities and efficacy of family support

Family support is commonly expressed through emotional reassurance, informational guidance, and practical assistance (14). Emotional support includes empathy, encouragement, and reassurance, which may alleviate anxiety and depressive symptoms. Informational support involves sharing knowledge about HBV transmission, treatment options, and long-term management, thereby facilitating informed decision-making and enhancing confidence in medical care. Practical support is reflected in assistance with healthcare visits, medication management, and daily activities (15). Empirical studies consistently demonstrate that adequate family support is associated with improved psychological well-being, greater social integration, and better disease management outcomes among people living with hepatitis B^16^. Individuals reporting stronger family support tend to exhibit higher levels of treatment adherence and overall quality of life.

### 2.3 Cultural nuances in family support

The form and impact of family support are shaped by sociocultural contexts (16). In many East Asian settings, family structures emphasize interdependence, collective responsibility, and filial obligation, which may enhance emotional and material support for individuals with chronic illness (17). In contrast, Western contexts often place greater emphasis on individual autonomy, with family support more frequently oriented toward fostering independence and self-management (18). These cultural differences may influence how HBV-related stigma is interpreted and negotiated within family systems, thereby shaping disease management trajectories. Clinical and public health interventions should therefore remain attentive to cultural variability and adapt family-centered approaches to local social norms and expectations (16, 19).

## 3 Hepatitis B–specific stigma ecology

### 3.1 Conceptual framework of health-related stigma

Health-related stigma is commonly conceptualized as a multilevel social process encompassing public stigma, self-stigma, and structural stigma (20). Public stigma refers to socially shared stereotypes and prejudicial attitudes toward individuals with a particular health condition. Self-stigma describes the internalization of these negative beliefs, potentially resulting in diminished self-worth and anticipated discrimination. Structural stigma denotes discriminatory norms, institutional practices, or policy arrangements that systematically disadvantage affected individuals.

The Health Stigma and Discrimination Framework provides an integrated perspective on how labeling, stereotyping, separation, and status loss operate across interpersonal, community, and institutional levels (21–23). This framework is useful for examining stigma in chronic infectious diseases, including hepatitis B. However, although the structural dimensions of stigma may be comparable across health conditions, the specific drivers, social meanings, and relational consequences of stigma are disease-dependent (24, 25). In the case of hepatitis B, transmission patterns, historical policy practices, and sociocultural interpretations of contagion and heredity generate distinctive stigma pathways that require focused analysis.

### 3.2 Conceptual distinction between stigma and discrimination

While stigma and discrimination are often used interchangeably in public discourse, they represent analytically distinct constructs. Stigma refers to the social process through which individuals are labeled, stereotyped, and socially devalued. Discrimination, in contrast, denotes the behavioral or institutional enactment of stigma through exclusion, unequal treatment, or policy restriction. In the context of hepatitis B, stigma shapes perceptions of contagion, responsibility, and moral status, whereas discrimination manifests in employment screening, service denial, and social exclusion. Distinguishing these constructs is essential for clarifying mechanisms and aligning intervention strategies at relational and structural levels (26).

### 3.3 Transmission pathways, reproductive structures, and intimate family dynamics

In many high-prevalence settings, hepatitis B is predominantly acquired through perinatal mother-to-child transmission, conferring a distinctive intergenerational dimension to HBV-related stigma (27). Unlike most non-communicable chronic diseases, HBV infection is often embedded within family transmission histories and intertwined with notions of lineage, responsibility, and familial continuity. Qualitative studies indicate that infection histories within kin networks may shape disclosure practices, attribution of responsibility, and emotional dynamics among family members (16, 28).

Concerns about vertical transmission may persist even in contexts where prevention programs are widely available. In settings with limited public understanding of transmission mechanisms, vertical transmission may be misinterpreted as caregiving failure or moral deficiency, thereby intensifying stigma within the family sphere (29, 30).

Beyond intergenerational transmission, hepatitis B carries implications for marriage and reproduction. In certain sociocultural contexts, infection identity may influence marital negotiations and partner selection, placing individuals under pressure regarding disclosure timing and relationship stability (4, 31, 32). Although prevention of mother-to-child transmission is highly effective, some families continue to express reservations about childbearing among people living with HBV, often rooted in misconceptions rather than current medical evidence. In some settings, women living with HBV may experience disproportionate scrutiny due to gendered expectations surrounding maternal responsibility and offspring health.

Taken together, transmission pathways and reproductive structures embed hepatitis B within core domains of family decision-making and intimate relationships. In this context, stigma reshapes disclosure strategies, relational trust, and patterns of family support. Understanding these mechanisms is essential for developing public health and nursing interventions that integrate medical counseling with relational and gender-sensitive family support.

### 3.4 Structural discrimination and persistent identity labeling

Beyond interpersonal and intimate relational dynamics, hepatitis B–related stigma is institutionalized and prolonged through structural and policy frameworks. In China and other high-prevalence settings, employment screening policies historically required HBV testing as a condition for entry into certain occupations, resulting in systematic exclusion from civil service, education, and service sectors. Policy reforms were implemented after 2007, and national regulations in 2010 prohibited employment restrictions based solely on HBV infection status (33, 34). Nevertheless, subsequent research suggests that, despite formal policy revisions, people living with HBV continue to report implicit discrimination and qualification scrutiny in hiring and career advancement (35, 36). These findings indicate that risk-based narratives institutionalized through earlier regulatory practices may persist beyond policy reform.

Concurrently, the biomedical classification of individuals as “Infection identity” has evolved socially into a durable identity label. Qualitative studies in China and migrant communities document ongoing concern about long-term social marking, even when viral replication is suppressed or individuals are asymptomatic (28). This identity stabilization reinforces anticipatory stigma and may encourage concealment of infection identity in employment and social settings.

Structural discrimination and persistent identity labeling do not operate in isolation. Through their effects on employment opportunities, income stability, and social mobility, they may indirectly reshape intra-family resource allocation and role distribution. Empirical research suggests that occupational restriction and economic insecurity can reduce individual bargaining power within households, translating institutional exclusion into relational strain (37, 38). HBV-related stigma therefore operates as a multilevel process: generated through institutional histories, materialized through discrimination and labeling, and reproduced within family relationships.

### 3.5 Multilevel psychological and behavioral consequences of HBV-related stigma

The consequences of hepatitis B–related stigma extend beyond emotional distress and emerge through the interaction of transmission narratives, reproductive pressures, and structural exclusion. Individuals situated within environments that frame HBV infection as a persistent risk must navigate ongoing decisions regarding disclosure and concealment. These decisions shape communication patterns, support-seeking behaviors, and family interactions.

Studies consistently demonstrate associations between HBV-related stigma and anxiety, depressive symptoms, and reduced quality of life (39). Research in Chinese populations indicates that perceived stigma is negatively correlated with psychological well-being and social adaptability, particularly in the early years following diagnosis (40).

Beyond psychological burden, stigma influences behavioral trajectories. Anticipated discrimination in healthcare, workplace, or community settings may lead to delayed screening, reduced follow-up, or fluctuations in treatment adherence (41). Qualitative research across diverse contexts shows that people living with chronic hepatitis B frequently engage in concealment and social withdrawal due to anticipated negative reactions. Survey evidence further indicates that greater secrecy about HBV status and stronger privacy needs are associated with higher perceived stigma (42).

Collectively, these findings suggest that HBV-related stigma functions as a relational and behavioral reconfiguration process. Through institutional risk narratives, social stereotyping, and family interaction patterns, individuals adjust disclosure strategies, support-seeking behaviors, and healthcare engagement. Hepatitis B stigma is therefore not merely a source of psychological distress but a multilevel social mechanism shaping disease management trajectories and family support dynamics (43).

## 4 Interaction between family support and HBV-related stigma

### 4.1 Family as the relational structure of stigma experience

HBV-related stigma does not operate as an isolated social label imposed directly on individuals; rather, it acquires concrete meaning within specific relational structures. Cross-cultural studies have consistently shown that in high-prevalence settings, initial disclosure following diagnosis most often occurs within the family. This sequence reflects not merely emotional proximity but the structural primacy of the family in the socialization of illness (28). At this early stage, family members’ interpretations of transmission risk, responsibility, and future life planning constitute the patient’s first social mirror of infection identity.

Within this relational context, the family both absorbs dominant societal risk narratives and reinterprets them. When HBV is framed in medicalized terms—as a manageable chronic condition—the infection identity tends to become de-moralized, reducing shame and internalized stigma. In contrast, when infection is embedded in narratives of intergenerational burden or reproductive risk, silence and caution often evolve into stable interaction patterns (44). Stigma, therefore, is not simply reproduced within the family but becomes concretized through ongoing interaction.

Moreover, sustained negotiation over whether and how to disclose infection to others shapes variations in stigma visibility. Qualitative evidence suggests that such negotiation may extend over years, influencing career decisions, marital arrangements, and broader social participation (45, 46). In this sense, the family functions not only as a source of support but as the relational conduit through which macro-level social structures are translated into lived experience. The organization of family interaction ultimately determines the diffusion boundaries and persistence of stigma in subsequent social encounters (16, 47).

### 4.2 Identity organization and repositioning within family structure

The label “Infection identity” is often associated with persistent risk in broader social discourse. Yet its practical meaning in everyday life is not determined unilaterally by external labeling; rather, it is organized, emphasized, or attenuated within family interaction structures. Evidence suggests that even when virological indicators remain stable or individuals are asymptomatic, infection history may be repeatedly framed as a risk reference point in family discussions—particularly in matters related to marriage, reproduction, and intergenerational planning (44). Under such conditions, the illness shifts from a medical fact to a relational variable embedded in decision-making.

Cross-cultural qualitative studies further indicate that when family members adopt a medicalized framing of HBV and define it as a manageable chronic condition, the salience of the infection identity within family interaction tends to decline (28). In these contexts, the disease is repositioned within the background of routine health management rather than functioning as a defining feature of relational roles. This process of normalization does not deny the presence of illness; instead, it redistributes its weight within the relational structure, reducing its dominance over family roles and decisions (48).

In this sense, Infection identity is not static but continuously positioned through interaction. The way families organize the meaning of infection determines the visibility and relational weight of the identity in daily life. In some families, infection remains a recurring focal topic; in others, it gradually becomes one component of health management. These variations suggest that stigma is shaped not solely by external social environments but also by internal narrative structures within the family (49, 50).

Family support and identity labeling thus exist in a dynamic tension. Support may weaken the moralized connotations of infection through knowledge reconstruction, yet repeated emphasis on risk can also reinforce the persistence of identity salience. Within this tension, the social meaning of HBV stigma is continually adjusted and renegotiated.

### 4.3 Institutional pressure and risk redistribution within family systems

Unlike individual-level shame or internalized stigma, structural discrimination often operates indirectly, entering family life through institutional practices such as employment restrictions, school screening policies, and social exclusion. In several high-prevalence settings, historical health examination requirements and occupational entry barriers have established durable associations between infection identity and employment opportunities. Even when formal policies evolve, collective memory and risk narratives may continue to exert influence (51, 52).

When individuals encounter opportunity constraints in the broader social sphere, the consequences frequently manifest first as adjustments in family roles. Changes in economic contribution may reshape internal decision-making dynamics, while occupational uncertainty can heighten family sensitivity toward disease management. Institutional exclusion thus becomes concretized within everyday discussions of responsibility allocation and risk management (45).

This process does not always generate overt conflict. Some families adopt protective strategies to buffer external pressures, including restricting information disclosure, avoiding perceived high-risk social contexts, or reinforcing caregiving roles. In certain situations, such protective reorganization may sustain family stability; in others, it may intensify the visibility of infection identity, turning the disease into a persistent reference point in household decision-making (28). Structural discrimination therefore does not operate as a linear external force. It is absorbed, reorganized, and redistributed within family systems. Through this mediation process, institutional pressure becomes embedded in relational structures, allowing stigma to persist as a socially conditioned variable rather than a singular event. This dynamic underscores the central position of the family within the ecology of HBV stigma: it both receives structural influence and shapes the individual’s re-entry into broader social space (33, 53).

### 4.4 The regulatory position of the family in the ecology of HBV stigma: an integrative perspective

HBV-related stigma does not move in a linear trajectory from social structure to individual experience. Rather, it undergoes continuous interpretation, reorganization, and redistribution within the family as a relational system. Disclosure negotiation shapes initial visibility, identity organization influences ongoing salience in daily life, and institutional pressure—mediated through family processes—affects its long-term embedding in economic and role structures.

The family therefore functions neither as a passive support resource nor as a simple counterforce to stigma, but as a dynamic regulatory node. In certain contexts, medicalized interpretation, emotional reassurance, and risk reframing may attenuate the social intensity of infection identity. In others, responsibility attribution, restricted information flow, or protective silence may inadvertently reinforce its persistent visibility. Through these relational dynamics, families can either constrain the diffusion boundaries of stigma or sustain its social meaning over time.

This interaction among relational, structural, and behavioral dimensions suggests that the impact of HBV stigma extends beyond individual psychological burden and should be understood within a multilevel social process. By absorbing societal discourse, organizing identity meaning, and shaping behavioral choices, the family occupies a pivotal analytical position in the ecology of HBV stigma (54). Recognizing this regulatory role provides a structured foundation for subsequent family-centered and nursing-led intervention frameworks.

## 5 Reconfiguring stigma pathways within family systems

### 5.1 Disclosure negotiation: structural regulation of stigma visibility

Within the operation of the HBV stigma pathway, disclosure is not a singular behavioral choice but a regulatory node that shapes the social visibility and diffusion boundaries of infection identity. The context in which infection information is communicated, the language through which risk is framed, and the actor who leads the interpretation collectively influence how stigma circulates within relational networks (16, 35).

The central issue in disclosure negotiation is not merely whether infection is revealed, but how identity is organized within relational structures. When disclosure occurs in the absence of medicalized explanation or under exaggerated risk imagination, emotional responses often precede rational assessment, giving rise to enduring interaction patterns such as silence, caution, or overprotection (35). Although such strategies may temporarily reduce external conflict, they can heighten identity salience over time, positioning infection as a persistent reference point in social participation and role allocation (45).

Intervention, therefore, should not be reduced to encouraging disclosure itself. Rather, it involves restructuring the risk interpretation framework embedded in disclosure negotiation. Through systematic risk communication training, scenario-based dialogue rehearsal, and role-position analysis, healthcare teams can support cognitive preparation prior to disclosure while guiding family members toward medicalized framing of infection (42). Such structural support allows disclosure to function as a point of relational recalibration rather than a trigger for stigma consolidation.

The stability of disclosure negotiation remains conditioned by the institutional environment. Where social discrimination retains tangible presence, families are more likely to adopt protective silence. Disclosure-oriented interventions must therefore be aligned with effective anti-discrimination enforcement and public awareness initiatives to prevent inadvertent exposure to structural risk. In this sense, disclosure support is not an adjunct psychological service but a critical entry point for reconfiguring the stigma pathway by regulating identity visibility.

At the level of practice, disclosure negotiation can be operationalized as structured support modules embedded within routine follow-up and chronic disease management systems. These modules may include pre-disclosure risk communication preparation, simulated dialogue exercises, and probability-based risk explanation training. Nursing-led communication support is particularly positioned to prevent emotional amplification as infection information enters family systems. Outcomes may be dynamically assessed through indicators such as post-disclosure psychological stability, family interaction quality, and social participation trajectories, thereby transforming disclosure support from episodic advice into a sustained relational intervention component.

### 5.2 Identity organization and relational repositioning

Within the ongoing operation of the HBV stigma pathway, infection identity functions as a relational variable rather than a fixed label. Whether infection becomes a central reference point in family decision-making and role allocation depends less on external societal labeling than on how its meaning is organized within family discourse.

When infection is embedded in narratives of intergenerational responsibility, reproductive risk, or long-term vulnerability, identity salience tends to remain elevated. Even in the presence of stable virological indicators or favorable clinical status, infection history may be repeatedly invoked during key family discussions, transforming a medical condition into a structural parameter of relational life. This persistent referencing mechanism can intensify perceived social sensitivity and shape self-evaluation processes (55).

Empirical findings suggest that when infection identity is moralized or framed primarily through risk within family interaction, individuals are more likely to experience sustained anxiety and depressive symptoms. Elevated identity salience may heighten concerns about relational stability and social evaluation, contributing to emotional exhaustion and avoidance behaviors. In certain contexts, such pressure may erode treatment confidence and weaken long-term adherence, shifting disease management from active engagement to passive maintenance. By contrast, when families adopt a medicalized framing and position HBV as a manageable chronic condition, the relational weight of infection declines. The disease becomes integrated into routine health management rather than serving as a defining relational marker. Such repositioning supports more stable self-concept, reduces psychological burden, and strengthens adherence trajectories (46, 56).

Intervention, therefore, should focus not on erasing labels but on restructuring internal meaning organization within the family system. Through systematic knowledge reconstruction and narrative reframing, infection can shift from a moralized risk symbol to a health-management status, thereby lowering identity salience and buffering emotional fluctuation and behavioral withdrawal at the relational level. Mechanistically, identity organization shapes both the temporal persistence of stigma and the trajectory of psychological stability and disease management (57). Regulating the relational distribution of identity weight offers a structural pathway to mitigate anxiety and depressive risk while promoting sustained treatment engagement.

At the implementation level, relational repositioning may be advanced through family-participatory education programs and sustained narrative reinforcement mechanisms. Unlike one-time informational sessions, these interventions emphasize repeated medical framing during long-term follow-up and reaffirmation at critical family decision points. Incorporating internalized stigma assessment and adherence indicators into routine management allows dynamic monitoring of changes in identity salience. Such sustained structural intervention gradually reduces the centrality of infection within family systems, supporting stable psychological states and consistent treatment behavior (58).

### 5.3 Institutional pressure, family mediation, and risk redistribution

HBV-related stigma does not arise solely within relational interaction; it remains embedded in institutional environments. Employment screening, occupational entry restrictions, and broader practices of social exclusion confer persistent risk meaning on infection identity at the structural level. Such institutional risk does not remain external but is absorbed and reorganized within family systems, where it becomes translated into role arrangements and resource allocation decisions (59).

When individuals face constrained opportunities or economic uncertainty due to infection identity, family responsibility structures often adjust accordingly. Shifts in income contribution, occupational planning, and prioritization of future goals may elevate infection as a key reference point in household decision-making. Institutional pressure rarely appears as overt conflict; instead, it becomes incorporated into everyday risk management discussions, transforming external exclusion into internal decision logic (53).

This mediation process carries long-term implications. Families may adopt protective strategies—such as cautious disclosure or modified career pathways—to buffer structural exclusion and preserve stability. Yet persistent institutional risk can simultaneously increase the relational weight of infection identity, making it an implicit premise of economic and life planning (43).

Institutional uncertainty may also intensify financial anxiety and risk perception, indirectly influencing disease management. When family resources are redirected toward coping with structural pressures, treatment behavior may be shaped by both material constraints and psychological burden, leading to fluctuations in adherence.

Intervention, therefore, must extend beyond intrafamilial communication to address institutional contexts and mediation pathways simultaneously. Strengthening anti-discrimination enforcement, improving transparency in social protection systems, and establishing resource-navigation support can reduce the continuous structural input of institutional pressure into family systems. Integrating institutional risk assessment into routine disease management further enables early identification of economic instability that may compromise sustained adherence. Mechanistically, institutional pressure influences the relational distribution of identity weight through family mediation. Regulating this pathway can help prevent the internal reproduction of external exclusion and support more stable role structures and long-term treatment engagement (60, 61).

### 5.4 The healthcare system and nursing as regulatory hubs in stigma pathway modulation

Across the mechanisms of disclosure negotiation, identity organization, and institutional pressure mediated through families, the healthcare system constitutes a central institutional arena for regulating the HBV stigma pathway. Within this arena, nursing occupies a position of structural advantage due to its sustained involvement throughout the continuum of disease management (62). Unlike episodic clinical encounters, nursing practice is embedded in long-term follow-up, health education, and routine communication. This continuity positions nurses as the most consistent professional contact for patients and their families. Such sustained engagement not only facilitates the transmission of medical knowledge but also shapes how infection identity is interpreted and how risk narratives are organized within relational contexts (63).

During disclosure processes, nursing participation in risk communication can introduce medicalized framing at the moment infection identity enters family systems, thereby influencing its social visibility. In the maintenance phase, repeated reinforcement of scientific understanding through follow-up and education can reduce identity salience in family interaction. When institutional pressures are introduced, nurses may serve as connectors to resources and policy guidance, assisting families in interpreting external risks and adjusting coping strategies to prevent the internalization of structural exclusion. Importantly, nursing systems often bridge clinical and community settings, integrating individual treatment management with public health education responsibilities. This cross-sector positioning enables nursing to function as a linkage node between healthcare institutions, family systems, and broader social structures (64, 65).

However, existing interventions addressing HBV stigma frequently remain confined to individual psychological support or advocacy initiatives, with limited integration of family-based regulatory mechanisms into routine disease management pathways. Embedding disclosure support, relational repositioning, and institutional risk assessment into nursing workflows can enable healthcare systems to assume a sustained regulatory function rather than offering episodic guidance (66).

From a pathway reconfiguration perspective, healthcare institutions provide structural legitimacy, while nursing operationalizes modulation through relational continuity. This structural positioning r*ender*s nursing indispensable within comprehensive HBV stigma intervention frameworks.

### 5.5 Public health context and evidence boundaries

Although this review proposes a family-centered framework for reconfiguring HBV stigma pathways, the current evidence base presents several limitations. Much of the literature on disclosure negotiation, identity organization, and institutional mediation relies on qualitative inquiry or cross-sectional designs, limiting the capacity to delineate dynamic transitions and causal sequencing across stages of the pathway (67).

Family structures and cultural norms vary substantially across contexts, shaping identity salience and risk interpretation in distinct ways. Disclosure practices and familial responses may therefore differ across institutional environments, requiring contextual adaptation when translating intervention frameworks into diverse settings (43).

Institutional variability further complicates implementation. Even where anti-discrimination principles are formally established, inconsistencies in enforcement may influence risk perception among patients and families. Such discrepancies between policy and practice can constrain the effectiveness of family-centered interventions.

From a public health perspective, HBV stigma emerges from multilevel interactions among structural, relational, and behavioral processes rather than from isolated individual behaviors. The sustainability of family-centered strategies depends on alignment with institutional protections and broader societal awareness (68–70). Recognizing these contextual and evidentiary boundaries supports more cautious and context-sensitive application of intervention models across different public health environments.

## 6 Conclusion

The persistence of HBV-related stigma indicates that it extends beyond an individual psychological phenomenon and is embedded within broader social structures and relational systems. Strategies centered solely on individual cognition or public awareness campaigns are unlikely to address the deeper mechanisms through which stigma is sustained. Positioning the family at the core of both analysis and intervention challenges the tendency to reduce stigma to personal experience. Families function not only as sources of emotional support but also as arenas in which infection identity is constructed, interpreted, and redistributed. Within these relational structures, social risk becomes concretized, behavioral choices are shaped, and long-term disease management trajectories take form. From a public health perspective, HBV management should move beyond virological control and clinical indicators to engage with the mechanisms through which stigma is reproduced across social systems. Structural reduction of stigma requires coordinated alignment among healthcare institutions, family support processes, and institutional protections. A family-centered framework therefore represents not merely an incremental addition to existing strategies but a necessary expansion of the conceptual foundations of HBV management. This perspective provides a structured basis for more sustainable and integrated public health action.
